# Changes of microbial population and N-cycling function genes with depth in three Chinese paddy soils

**DOI:** 10.1371/journal.pone.0189506

**Published:** 2017-12-28

**Authors:** Huanhuan Wang, Xu Li, Xiang Li, Xinyu Li, Jian Wang, Huiwen Zhang

**Affiliations:** 1 Key Laboratory of Pollution Ecology and Environmental Engineering, Institute of Applied Ecology, Chinese Academy of Sciences, Shenyang, China; 2 Graduate University of Chinese Academy of Sciences, Beijing, China; The University of Akron, UNITED STATES

## Abstract

Microbial communities play critical roles in soil nitrogen (N) cycle; however, we have limited understanding of the distribution of N-cycling microbial groups in deeper soil horizons. In this study, we used quantitative PCR to characterize the changes of microbial populations (16S rRNA and 18S rRNA) and five key N-cycling gene abundances involved in N fixation (*nifH*), ammonia oxidation (*amoA*) by ammonia-oxidizing bacteria (AOB) and ammonia-oxidizing archaea (AOA), and nitrite reduction (*nirS* and *nirK*) along profiles (0–100 cm depth) of different paddy soils from three regions (Hailun, Changshu, Yingtan) across China from north to south. We found that most microbial and N-cycling functional genes significantly decreased with soil depth; however, AOA were enriched in deeper soil layers (20–40 cm). The abundances of microbial and N-cycling functional genes generally decreased by one to two orders of magnitude in the deeper horizons relative to topsoils. The AOA gene abundance was higher than that of AOB in the paddy soil profile, and the *nirS* and *nirK* abundances were dominant in topsoil and deeper soil, respectively. All N functional genes except AOA were more abundant in Changshu than Hailun and Yingtan. High abundances and low vertical changes of N-cycling genes in Changshu suggest more dynamic N-transformations in this region. Correlation analysis showed that soil properties and climate parameters had a significant relationship with N-cycling gene abundances. Moreover, the abundance of different N-cycling genes was affected by different environmental parameters, which should be studied further to explore their roles in N cycling for sustainable agriculture and environmental management.

## Introduction

Paddy ecosystems are essential components of agricultural systems, and support more than half of the world’s population [1]. Soil microorganisms play an important role in paddy ecosystem, and can significantly affect soil fertility and rice productivity by participating in decomposition processes and nutrient cycles, particularly N cycling [2–5]. Nitrogen is generally the most limiting nutrient for rice production, and microbiological processes, e.g. nitrogen fixation, nitrification and denitrification, were important in N cycling [6]. Biological nitrogen fixation can reduce atmospheric N_2_ to bioavailable NH_4_^+^, and provide natural N input to cultivated rice cropping systems. Nitrification and denitrification are key processes that determine the efficiency of fertilizer use by rice crop, N loss from rice paddy soils, environmental pollutions such as nitrate leaching and emission of nitrous oxide [7]. However, we have limited understanding of microbial community distribution and functional characteristics in subsoil (below 0.2 m), as most studies have focused on the top 0.2 m of the soil column. Previous studies using DNA fingerprinting [8–10], phospholipid fatty acid [11–13] and 16S rRNA Miseq sequencing [13, 14] have demonstrated that microbial community composition and diversity vary with soil depth, and microbial biomass declines exponentially with soil depth. However, few studies have attempted to comprehensively assess the change of microbial population and the abundance of nitrogen (N)-cycling function genes with soil depth.

Some key N-cycling genes encoded for enzymes involved in N-transformations including N-fixation (*nifH*), nitrification (bacterial and archaeal *amoA*), and denitrification (*nirS* and *nirK*) have been widely used to evaluate the functional abundance and diversity of soil microbial communities [15, 16]. Previous studies have indicated that the abundances of soil N-cycling genes are associated with soil properties [17, 18], soil type [19], and different N fertilizer regimes [20]. The abundances of N-cycling genes vary with soil depth, but the variability is different for different N-cycling genes. Leininger et al. (2006) found that both AOA and AOB gene abundances decreased with soil depth, but AOB *amoA* gene abundance decreased more strongly with depth than AOA at 0–40 cm soil depth [21]. However, in some wetland sites, the gene abundances of both AOA and AOB increased with soil depth at 0–30 cm depth, especially at high-ammonium sites [22]. Some studies found that *nirS* genes were more abundant than *nirK* genes [23], but other studies have shown the opposite trend [24]. These studies suggested that N-cycling genes require further investigation.

China is the world’s largest rice producer [25], and rice is planted in a large latitude range and on diverse soil types in China. In this study, we selected three typical paddy soils with distinct soil properties across a latitude gradient from northern to southern China to examine the shifts of microbial population and N-cycling gene abundance with soil depth (0–100 cm) in three different paddy soils with the q-PCR method, and to explain the environmental factors controlling this variation.

## Materials and methods

I state clearly that no specific permissions were required for these locations/activities, which are concerned about scientific study in these regions in China. I confirm that the field studies did not involve endangered or protected species.

### Soil sampling and characterization

The method of soil sampling and the determination of soil physicochemical properties have been previously described [12, 26]. Briefly, 90 soil samples were collected from Hailun (126°51′–126°58′ E, 47°31′–47°33′ N, 168–226 m elevation), Changshu (120°35′–120°40′ E, 31°30′–36°33′ N, 2–6 m elevation), and Yingtan (116°54′–116°56′ E, 28°10′–28°13′ N, 34–62 m elevation) in three rice production areas from north to south China after the late rice harvest (October–November 2014). Soil types were Hapli-Udic lsohumosols, Gleyic-Stagnic Anthrosols, and Argi-Udic Ferrosols for Hailun, Changshu, and Yingtan respectively, according to the Chinese Soil Taxonomy [12]. Annual precipitation and number of frost-free days per year at these three regions are as follows: 1795 mm annual precipitation and 264 frost-free days for Yingtan; 530 mm annual precipitation and 149 frost-free days for Hailun; and 1028 mm annual precipitation and 235 frost-free days for Changshu. In each region, five replicates were collected from five randomly selected sites (at least 3 km apart from each other). Within each site, soil samples were taken vertically at 0–10, 10–20, 20–40, 40–60, 60–80, and 80–100 cm soil depth. The characteristics of the sampling sites are shown in Table 1.

**Table 1:** Soil properties at different soil layers in three study sites.

Site	profile (cm)	Sand Clay %		pH	Total C (g kg^-1^)	Total N (g kg^-1^)	Soil moisture	DOC (mg kg^-1^)	NO_3_^-^-N (mg kg^-1^)	NH_4_^+^-N (mg kg^-1^)	C/N
**Changshu**	0–10	5.11	34.64	5.46	22.58	2.22	0.46	10.75	3.22	2.28	10.15
	10–20	3.29	36.47	6.55	16.48	1.68	0.34	13.28	1.21	1.48	9.82
	20–40	2.08	37.57	7.44	9.10	0.96	0.33	13.38	1.25	2.03	9.39
	40–60	1.41	36.28	7.51	5.90	0.62	0.30	14.11	0.94	1.80	9.49
	60–80	1.63	36.85	7.63	5.64	0.50	0.31	13.78	0.62	1.76	12.25
	80–100	1.11	42.15	7.41	6.30	0.60	0.35	13.07	0.55	2.89	10.60
**Hailun**	0–10	3.79	34.88	6.18	34.90	2.70	0.46	10.84	0.74	1.48	14.14
	10–20	3.60	34.07	6.62	33.04	2.50	0.41	11.76	0.35	2.00	14.29
	20–40	2.55	35.04	6.93	23.64	1.64	0.37	12.62	0.50	2.34	14.57
	40–60	2.05	36.61	6.74	16.52	1.10	0.34	13.28	0.64	1.21	15.07
	60–80	2.29	38.05	6.65	14.16	0.92	0.34	11.76	0.80	1.61	15.49
	80–100	1.99	38.53	6.57	10.02	0.74	0.30	14.01	0.95	1.58	13.58
**Yingtan**	0–10	34.55	43.85	4.71	14.50	1.52	0.39	12.16	2.42	1.37	9.54
	10–20	30.99	46.88	5.12	7.64	0.82	0.28	14.44	1.77	1.45	9.33
	20–40	27.27	50.95	5.43	3.64	0.42	0.27	14.70	1.15	1.78	8.77
	40–60	27.00	51.57	5.42	2.88	0.38	0.27	14.62	0.60	1.76	7.77
	60–80	26.65	51.69	5.24	2.18	0.32	0.28	14.39	0.87	1.78	6.88
	80–100	24.05	53.45	5.01	1.66	0.32	0.24	15.29	1.31	2.25	5.35

DOC, dissolved organic carbon; C/N, Total C: total N ratio.

### DNA extraction

Soil DNA was extracted from 0.5 g of freeze-dried soil samples using the FastDNA SPIN Kit for soil (MP Biomedicals, Solon, OH, USA), according to the manufacturer's instructions. The DNA quality was assessed in agarose gel (1%) and DNA quantity was measured with a Nanodrop spectrophotometer (NanoDrop, Wimmington, DE, USA). The extracted DNA was stored at −20°C for subsequent quantification of microbial population and functional genes.

### Quantitative PCR

The abundances of bacterial 16S rRNA gene, fungal 18S rRNA gene and N-cycling functional genes (*nifH*, AOA-*amoA*, AOB-*amoA*, *nirS* and *nirK*) were measured by qPCR using SYBR® Premix Ex *Taq*™ II (Takara, Japan) on a LightCycler® 96 Real-time PCR System (Roche, Switzerland). The qPCR reaction (20 μl) contained 10 μl of SYBR Premix Ex *Taq*™ II, 0.4 μl each of the forward and reverse primers (Table 2), 2 μl of template DNA and 7.2 μl of sterile ultrapure water. The PCR cycling conditions were as follows: 45 cycles of 95°C for 10 s, primer annealing temperature for 30 s (Table 2) and template extension at 72°C for 45 s.

**Table 2:** PCR primers used for the amplification of functional target genes.

Primer	Target gene	Sequence (5’-3’)	Annealing temperature (°C)	Reference
**Bacteria-341F**	16S rRNA	CCTACGGGAGGCAGCAG	55	[27]
**Bacteria-758R**	16S rRNA	CTACCAGGGTATCTAATCC	55	
**Fungal-FR1**	18S rRNA	AICCATTCAATCGGTAIT	50	[28]
**Fungal-FR390**	18S rRNA	CGATAACGAACGAGACCT	50	
**Arch-amoAF**	AOA *amoA*	STAATGGTCTGGCTTAGACG	53	[29]
**Arch-amoAR**	AOA *amoA*	GCGGCCATCCATCTGTATGT	53	
**amoa-1F**	AOB *amoA*	GGGGTTTCTACTGGTGGT	58	[30]
**amoa-2R**	AOB *amoA*	CCCCTCKGSAAAGCCTTCTTC	58	
**nifH-F**	*nifH*	AAAGGYGGWATCGGYAARTCCACCAC	60	[31]
**nifH-R**	*nifH*	TTGTTSGCSGCRTACATSGCCATCAT	60	
**nirS-cd3aF**	*nirS*	AACGYSAAGGARACSGG	58	[32]
**nirS-R3cd**	*nirS*	GASTTCGGRTGSGTCTTSAYGAA	58	
**nirK-FlaCu**	*nirK*	ATCATGGTSCTGCCGCG	57	[33]
**nirK-R3Cu**	*nirK*	GCCTCGATCAGRTTGTGGTT	57	

Note: S = G/C; K = G/T; Y = C/T; R = A/G; W = A/T.

To obtain the standard curves for qPCR assays, the bacterial 16S rRNA gene, fungal 18S rRNA genes and functional genes (AOA-*amoA*, AOB-*amoA*, *nifH*, *nirS* and *nirK*) were amplified with the primers listed in Table 2. The PCR products from each gene were purified with a Gel Extraction Kit (CW Biotech co, Beijing, China) and cloned into pMD19-T vector (Takara, Japan). Plasmids from the positive clones with the target gene insert were extracted with a PurePlasmid Mini Kit (CW Biotech Co., Beijing, China). The concentration of plasmid was determined on a NanoDrop 2000 spectrophotometer (NanoDrop Technologies, Wilmington, DE, USA), and used for the calculation of standard copy numbers. Ten-fold serial dilutions of plasmid in triplicate were used to generate a standard curve for each gene and to check the amplification efficiency. For each gene, a high amplification efficiency of 92–98% was obtained, the R^2^ values were >0.992 and no signal was observed in the negative controls. The copy numbers for each sample of soil DNA were calculated based on comparison with the standard curve.

### Statistical analysis

Statistical analyses of the effects of region and soil depth on microbial population and N-functional genes were conducted by two-way analysis of variance (ANOVA) using Tukey’s honest significant difference (HSD) multiple comparison with a *P* = 0.05 grouping baseline. All of the statistical analyses were performed with SPSS 16.0 (SPSS Inc., Chicago, IL, USA). Spearman correlation analysis was employed to determine the significant correlations between the abundances of microbial population and N-cycling genes and environmental parameters. The correlation coefficients were calculated and plotted using the "corrplot" package in R software (version 3.3.1). The probability level *P* < 0.05 was considered to be statistically significant.

## Results

### Abundance of bacteria and fungi

**Fig 1:**
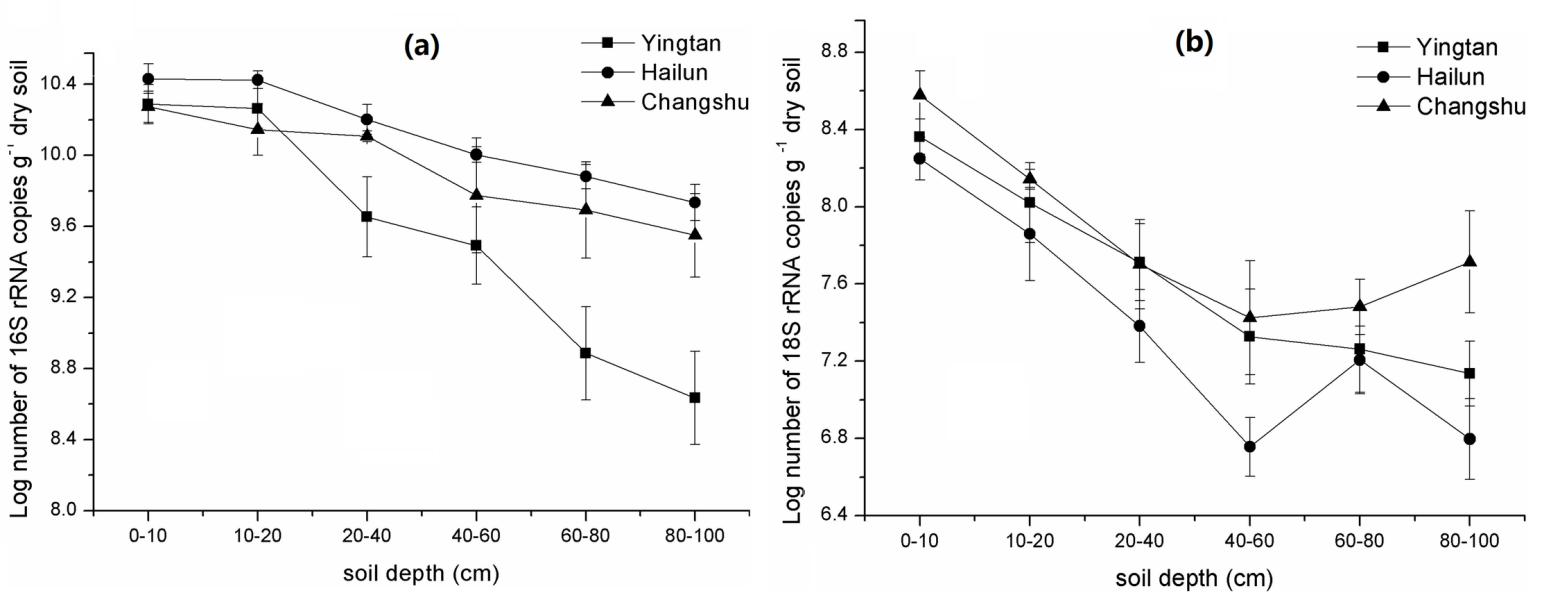
The abundances of bacteria 16S rRNA (a) and fungal 18S rRNA (b) genes at different depths in three regions.

The bacterial 16S rRNA gene copy numbers in all three regions ranged from 2.70 × 10^10^ to 4.31 × 10^8^ copies g^−1^ dry soil, which were more abundant than fungal 18S rRNA gene copy numbers, ranging between 3.56 × 10^8^ and 5.71 × 10^6^ copies g^−1^ dry soil (Fig 1A and 1B). The ANOVA revealed that soil depth, region, and their interaction all had significant effects on bacteria and fungi population (*P* < 0.001, Table 3). Bacterial 16S rRNA gene numbers slightly decreased at 0–20 cm soil depths, then significantly decreased at 20–100 cm soil depths in all three regions but especially Yingtan. The abundance of 16S rRNA gene throughout soil profile generally decreased in the order Hailun > Changshu > Yingtan. The bacterial 16S rRNA gene copy numbers in Changshu ranged from 1.88 × 10^10^ to 3.55 × 10^9^ copies g^−1^ dry soil, and reduced by 81.11% with depth. The ranges for Hailun and Yingtan were 2.70 × 10^10^ to 5.44 × 10^9^ and 1.94 × 10^10^ to 4.31 × 10^8^ copies g^−1^ dry soil, and decreased by 79.85% and 97.77% with depth, respectively. Fungal 18S rRNA gene copy numbers significantly decreased at 0–60 cm depths in the three regions, especially in the Hailun soil, and there were no significant differences among the 40–60, 60–80, and 80–100 cm depths. The abundance of 18S rRNA gene throughout soil profile generally decreased in the order Changshu > Yingtan > Hailun. The fungal 18S rRNA gene copy numbers in Changshu ranged from 3.56 × 10^8^ to 2.66 × 10^7^ copies g^−1^ dry soil, and reduced by 92.52% with depth. The ranges for Hailun and Yingtan were 1.78 × 10^8^ to 5.71 × 10^6^ and 2.30 × 10^8^ to 1.37 × 10^7^ copies g^−1^ dry soil, and decreased by 96.79% and 94.04% with depth, respectively. The abundances of both 16S rRNA and 18S rRNA genes were generally one to two orders of magnitude lower in the deeper horizons than the topsoils.

**Table 3:** Two-way ANOVA analysis of the abundances of bacteria, fungi and N-cycling functional genes at six soil depths in three sites each with five replicates (n = 90). The data in italics indicated that abundance of N-cycling gene was affected by soil depths, sites or their interaction.

		Bacteria	Fungi	AOA	AOB	*nifH*	*nirK*	*nirS*
**Region(R)**	F	105.661	44.205	89.032	17.792	3.902	428.767	172.615
	*P*	*<0*.*001*	*<0*.*001*	*<0*.*001*	0.054	*0*.*025*	*<0*.*001*	*<0*.*001*
**Depth(D)**	F	56.005	68.927	58.019	50.099	35.850	150.656	149.293
	*P*	*<0*.*001*	*<0*.*001*	*<0*.*001*	*<0*.*001*	*<0*.*001*	*<0*.*001*	*<0*.*001*
**Interaction** **(R×D)**	F	7.122	1.613	8.153	1.455	1.366	7.922	6.591
	*P*	*<0*.*001*	*0*.*027*	*<0*.*001*	0.392	*0*.*021*	*<0*.*001*	*<0*.*001*

*P* values (*P* < 0.05) are indicated in italics.

**Fig 2:**
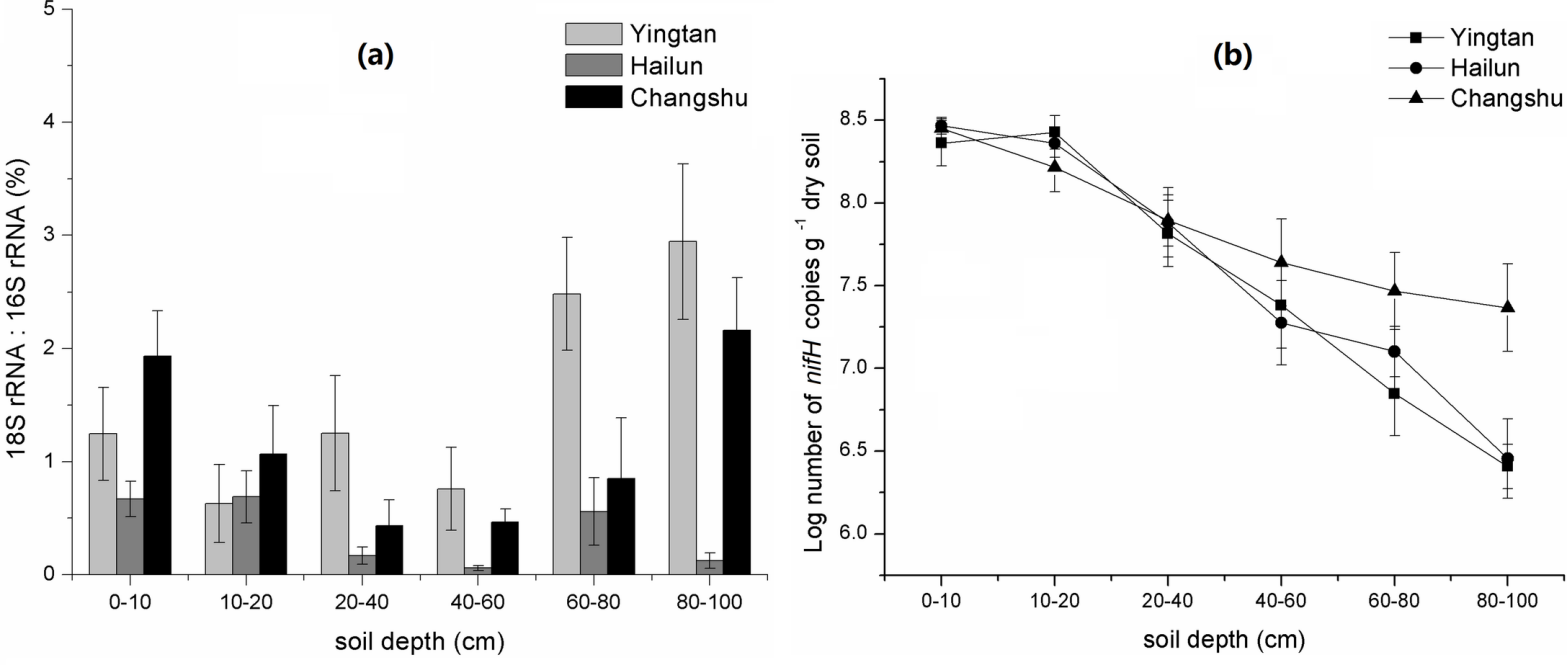
The ratios of bacteria 16S rRNA to fungal 18S rRNA gene copy numbers (a) and the abundances of *nifH* gene (b) at different depths in three regions.

The ratio of 18S rRNA to 16S rRNA gene abundance generally decreased at 0–60 cm depth and increased at 60–100 cm depth in the three regions (Fig 2A). At 0–60 cm soil depth, the ratios of 18S rRNA/ 16S rRNA decreased from 0.6–1.9% to 0.05–0.7%, and then the ratios gradually increased to 0.1–2.9% at 60–100 cm soil depth. According to the changes of 16S rRNA and 18S rRNA gene abundances with soil depth, the trends of variation in the ratio indicated that the abundance of 18S rRNA gene decreased more strongly than 16S rRNA gene at soil depths between 0 and 60 cm but the opposite occurred at 60–100 cm soil depth.

### N-cycling functional gene abundance

#### N-fixation

The *nifH* gene is widely used as a marker for molecular analysis of N-fixing bacteria. In this study, the *nifH* gene copy numbers varied from 3.16 × 10^8^ to 2.55 × 10^6^ copies g^−1^ dry soil across the three regions (Fig 2B). Region, depth, and their interaction all had significant effects on the abundance of *nifH* (Table 3). The *nifH* gene numbers significantly decreased with increasing soil depth in all three regions except for 0–20 cm soil depth in Yingtan soil, where the *nifH* gene number slightly increased. The *nifH* gene copy numbers in Changshu ranged from 2.83 × 10^8^ to 2.38 × 10^7^ copies g^−1^ dry soil, and reduced by 91.59% with depth. The ranges for Hailun and Yingtan were 2.78 × 10^8^ to 2.85 × 10^6^ and 3.16 × 10^8^ to 2.55 × 10^6^ copies g^−1^ dry soil, and decreased by 98.97% and 99.19% with depth, respectively. The abundance of the *nifH* gene generally decreased by one to two orders of magnitude with soil depth.

#### Nitrification

**Fig 3:**
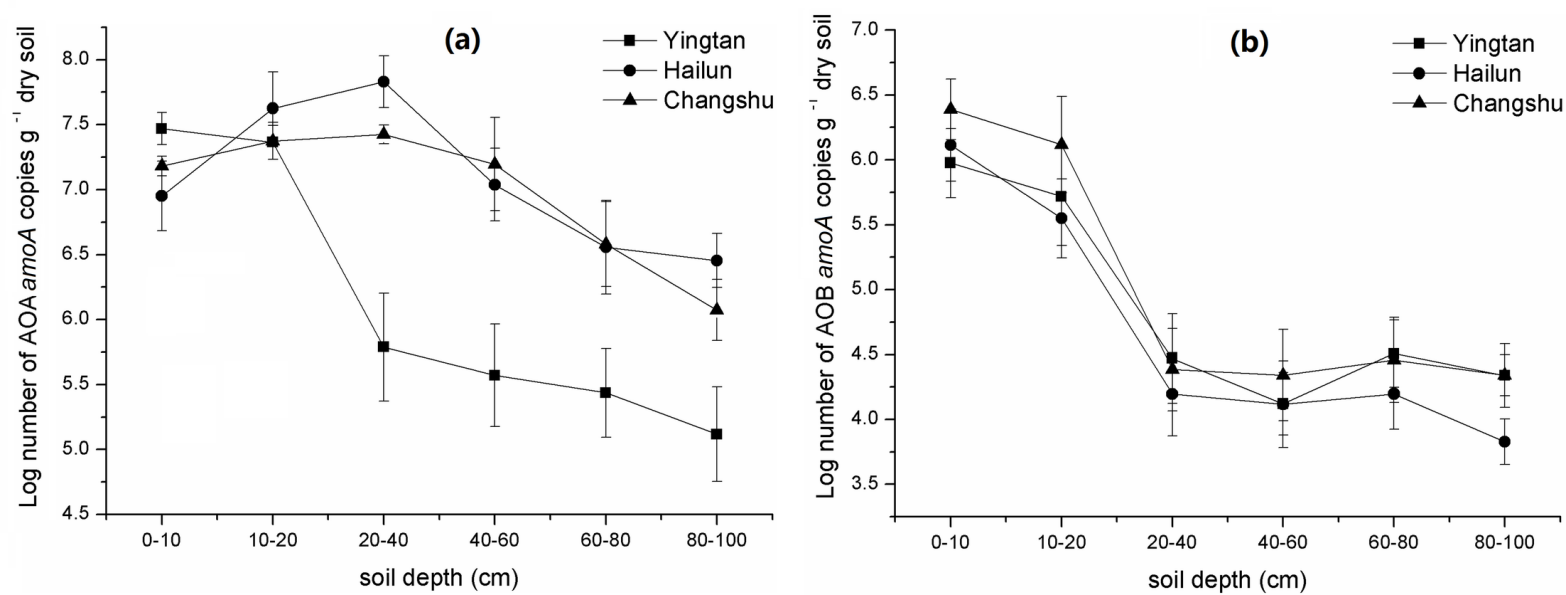
The abundances of ammonia-oxidizing bacteria (AOB) and ammonia-oxidizing archaea (AOA) *amoA* genes at different depths in three regions.

The AOA *amoA* gene copy numbers in all three regions, ranging from 6.20 × 10^7^ to 1.31 × 10^5^ g^−1^ dry soil, were higher than those of AOB *amoA* copy numbers, ranging between 2.44 × 10^6^ and 6.72 × 10^3^ copies g^−1^ dry soil (Fig 3A and 3B). The ANOVA revealed that region, depth, and their interaction all had significant effects on AOA *amoA* gene abundances (*P* < 0.001), but only depth had a significant effect on AOB *amoA* gene numbers (Table 3). The abundances of the AOA *amoA* gene significantly changed with soil depth with an increase at depths between 0 and 40 cm, followed by a decrease at depths between 40 and 100 cm, except for the AOA *amoA* gene in Yingtan, which generally decreased with increasing soil depth. The AOA *amoA* gene abundance peaked at 20–40 cm depth for Hailun and Changshu soils. The AOA *amoA* gene copy numbers in Changshu ranged from 2.65 × 10^7^ to 1.18 × 10^6^ copies g^−1^ dry soil, and reduced by 96.30% with depth. The ranges for Hailun and Yingtan were 6.20 × 10^7^ to 2.21 × 10^6^ and 2.10 × 10^7^ to 1.31 × 10^5^ copies g^−1^ dry soil, and decreased by 96.43% and 99.37% with depth, respectively. The AOB *amoA* gene copy numbers significantly decreased at depths between 0 and 40 cm depths (especially 20–40 cm depth) in all three regions, but there was no significant difference among the 40–60, 60–80, and 80–100 cm depths. The AOB *amoA* gene copy numbers in Changshu ranged from 2.44 × 10^6^ to 2.20 × 10^4^ copies g^−1^ dry soil, and reduced by 99.09% with depth. The ranges for Hailun and Yingtan were 6.72 × 10^5^ to 6.72 × 10^3^ and 4.33 × 10^5^ to 2.18 × 10^4^ copies g^−1^ dry soil, and decreased by 99.00% and 94.96% with depth, respectively. The abundance of both AOA and AOB *amoA* genes generally decreased by one to two orders of magnitude along soil depth gradients.

**Fig 4:**
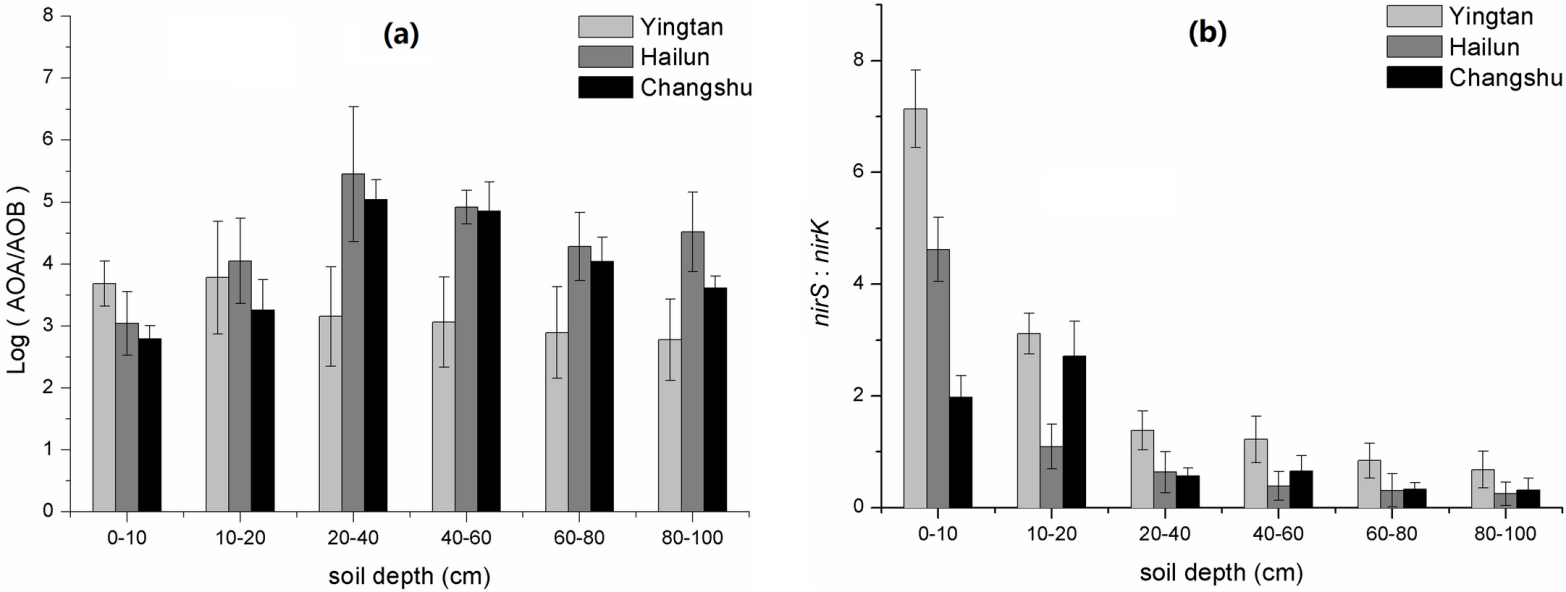
The ratios of AOA to AOB *amoA* gene copy numbers (a) and *nirS* to *nirK* gene copy numbers (b) at different depths in three regions.

The ratio change of AOA to AOB *amoA* gene abundances was similar with the change of AOA *amoA* gene abundance with soil depths (Fig 4A). The ratio increased at 0–40 cm soil depth, and then decreased at 40–100 cm soil depths in Hailun and Changshu soils, but the ratio in Yingtan generally decreased with increasing soil depth. The ratio peaked at 20–40 cm depth, and AOA *amoA* gene abundances were two to five orders of magnitude higher than AOB *amoA* gene in soil profiles.

### Denitrification

**Fig 5:**
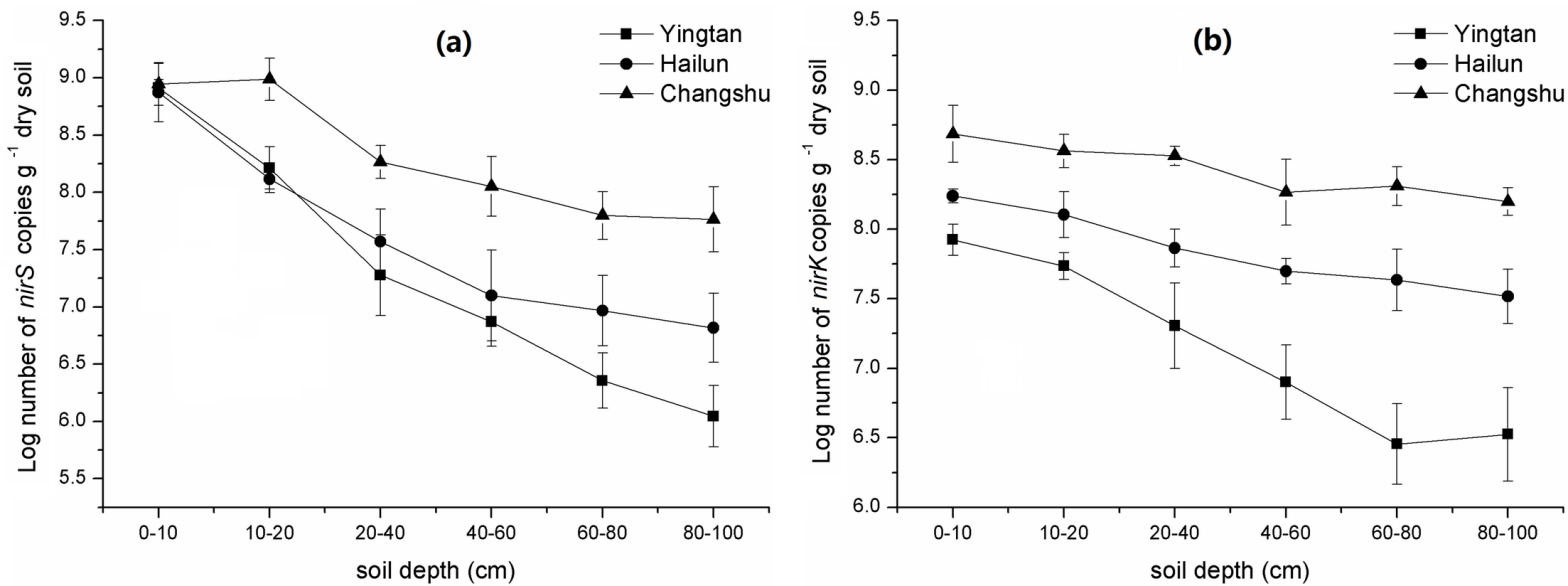
The abundances of *nirS* (a) and *nirK* (b) genes at different depths in three regions.

The *nirS* gene copy numbers ranged from 9.72 × 10^8^ to 1.11 × 10^6^ g^−1^ dry soil among the three regions and *nirK* gene abundances were from 4.85 × 10^8^ to 2.85 × 10^6^ g^−1^ dry soil (Fig 5A and 5B). Two-way ANOVA showed that soil depth, region, and their interaction all had significant effects on the *nirS* and *nirK* gene copy numbers (Table 3). The changes of *nirS* and *nirK* gene abundances with soil depth in all three regions showed a similar trend in which the copy numbers of both genes generally decreased with increasing soil depths. The abundances of both genes throughout soil profile generally decreased in the order Changshu > Hailun > Yingtan. The *nirS* gene abundances in Changshu ranged from 9.72 × 10^8^ to 5.79 × 10^7^ copies g^−1^ dry soil, and reduced by 94.04% with depth. The ranges for Hailun and Yingtan were 7.43 × 10^8^ to 6.57 × 10^6^ and 6.75 × 10^8^ to 1.11 × 10^6^ copies g^−1^ dry soil, and decreased by 99.11% and 99.83% with depth, respectively. The *nirK* gene abundances in Changshu ranged from 4.85 × 10^8^ to 1.84 × 10^8^ copies g^−1^ dry soil, and reduced by 62.06% with depth. The ranges for Hailun and Yingtan were 1.73 × 10^8^ to 3.29 × 10^7^ and 8.39 × 10^7^ to 2.85 × 10^6^ copies g^−1^ dry soil, and decreased by 80.98% and 96.60% with depth, respectively. The abundance of the *nirS* gene generally decreased by one to two orders of magnitude along soil depth gradients. In contrast, the abundance of the *nirK* gene generally decreased by one order of magnitude along soil depth gradients.

The ratios of *nirS* to *nirK* gene abundances generally decreased with increasing soil depths, except for the 10–20 cm depths in Changshu soil, where the ratio slightly increased (Fig 4B). The ratio ranged from 9.04 to 0.24. The *nirS* gene abundances were higher than those of the *nirK* gene at depths between 0 and 20 cm in Changshu and Hailun soils and between 0 and 60 cm in Yingtan soil. However, *nirK* gene abundances were higher than those of the *nirS* gene at depths between 20 and 100 cm in Changshu and Hailun soils and between 60 and 100 cm in Yingtan soil.

### Relationships between microbial population, functional gene abundances and environmental parameters

**Fig 6:**
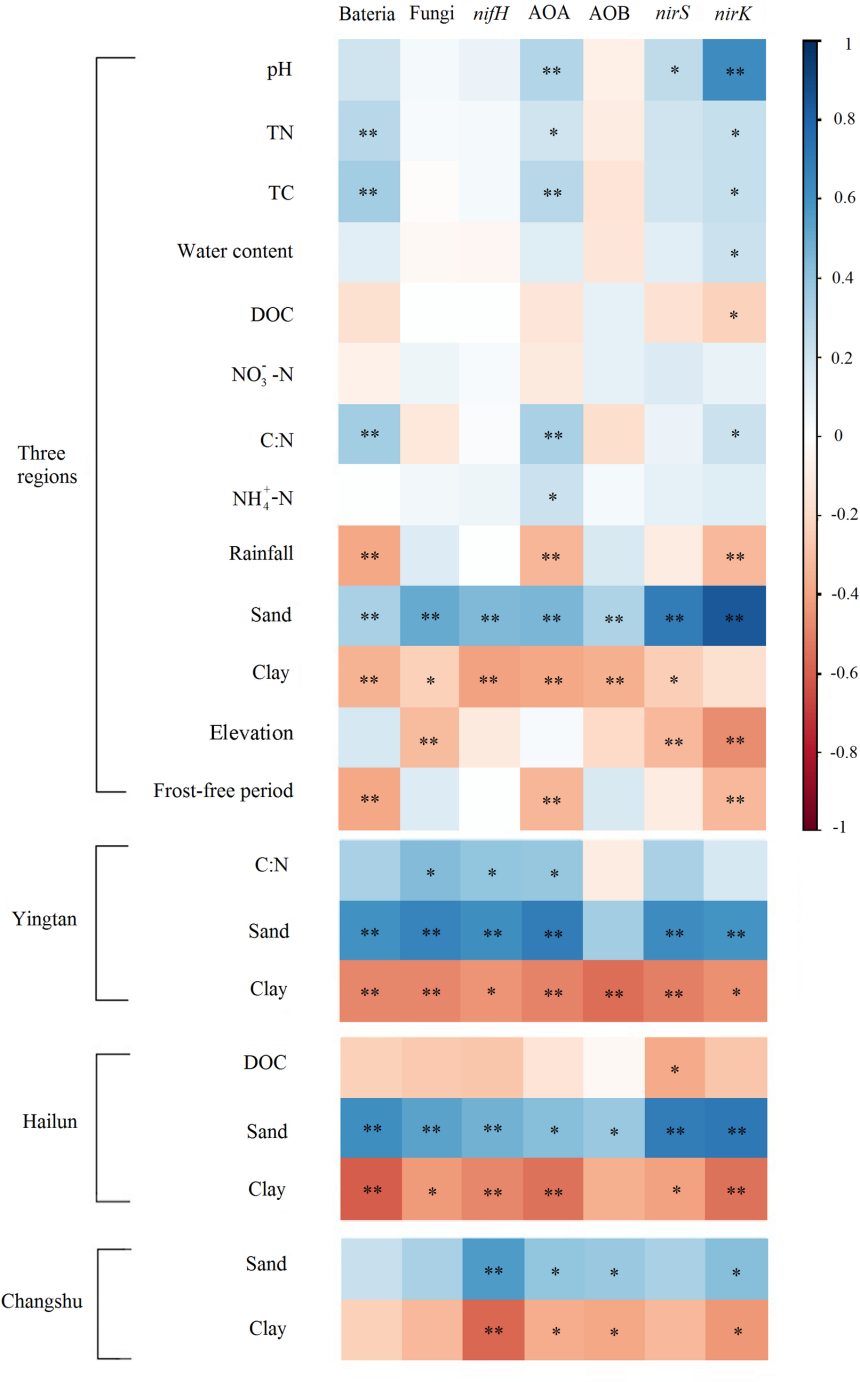
Correlogram representing spearman’s correlation coefficient rank between environmental parameters and abundances of microbial and of N cycling genes. The correlation coefficients ranging from negative to positive are indicated by color intensity changing from red to blue. *P < 0.05, **P < 0.01.

Spearman’s correlation coefficients were used to compare environmental parameters and the abundances of bacterial 16S rRNA, fungal 18S rRNA and N-cycling functional genes. In Fig 6, all environmental parameters were shown in the correlation analysis across the three regions, but for the correlation analysis of single regions, only the environmental factors that significantly correlated with the abundances of bacterial 16S rRNA, fungal 18S rRNA or N-cycling functional genes are presented. The correlation analysis across three regions, integrating the influences of depth and region, revealed that soil physical structure (sand and clay) correlated with all microbial and N-cycling functional gene abundances, except *nirK* and clay. In addition to soil physical structure, both *nirS* and *nirK* gene abundances were significantly correlated with elevation and pH. Furthermore, *nirK*, AOA and 16S rRNA gene abundances had more significant correlations with environmental parameters than *nirS*, AOB and 18S rRNA, and were positively correlated with total N, total carbon (C), C/N ratio and negatively correlated with annual precipitation and frost-free period. The 18S rRNA gene abundances were negatively correlated with elevation. In addition, *nirK* gene abundance was also correlated with soil water content and dissolved organic carbon (DOC) concentration, and AOA gene abundance positively correlated with nitrate concentration. For the correlation analysis of single regions, results similar to the total correlation analysis across three regions were shown (soil physical structure (sand and clay) correlated with most of the microbial and N-cycling functional gene abundances). In addition to soil physical structure, the changes of *nirS* gene abundances along the soil profile negatively correlated with DOC concentration, and the abundance changes of 18S rRNA, *nifH* and AOA genes were significantly positively correlated with the C/N ratio.

## Discussion

### Shifts in the abundances of bacteria and fungi along the soil profile across the three regions

Both bacteria and fungi were one to two orders of magnitude higher in the upper 10 cm of soil than at 1 m depth. Decreasing microbial biomass with soil depth has been previously observed in similar studies [34–36]. Throughout the soil profiles, we observed significant differences in nutrient concentrations (C and N), soil moisture, pH, and soil texture with soil depth (Table 1). Both bacteria and fungi in surface soils are likely to have greater nutrient resources and environmental conditions that favor their growth. Although both bacterial and fungal abundance decreased with soil depth, fungal abundance decreased more dramatically than bacteria at depths between 0 and 60 cm. This is similar to the findings of previous study about community structure that indicated fungal communities were more strongly influenced by soil depth than bacterial communities [37]. We observed that the change of fungi abundance along soil depth was significantly correlated with soil C/N ratio, and the results are broadly consistent with work by Lauber [38], who found that fungal community was most closely associated with soil nutrient status and the soil C/N ratio. At the large scale across the three regions, the abundances of both bacteria and fungi were significantly different among the three regions. This supports the findings of other studies that soil type or site conditions are also the determining factors of changes in bacterial and fungal communities [38–41].

### Shifts in the abundances of N-cycling functional groups along the soil profile across the three regions

In terrestrial ecosystems, the inputs of N to soil mainly depend on biological N fixation. Nitrogen-fixing bacteria use nitrogenase enzymes to catalyze this process. The *nifH* gene is widely used as a marker for molecular analysis of N-fixing bacteria [42]. In our study, the abundance of the *nifH* gene substantially declined with depth in the three soil profiles, which is consistent with the findings of other studies [18, 43]. Moreover, we observed that the abundance changes of the *nifH* gene with soil depth were significantly correlated with soil C/N ratio. Some studies have previously indicated that C/N is an important factor influencing the abundance of N-fixing bacteria [44, 45]. The increase in C/N ratio that C-rich and N-depleted conditions might provide a competitive advantage for N-fixing bacteria.

Nitrification, the microbial oxidation of ammonia to nitrite and nitrate, is a key process in the global cycling of N. The first and rate-limiting step is catalyzed by the enzyme ammonia monooxygenase (AMO), which exists in both AOB and AOA [46]. In our study, the abundance of AOA was greater than that of AOB in all the soil samples, and only AOA abundances were significantly positively correlated with the ammonium content (substrate of ammonia oxidation), which suggested that AOA are more important nitrifiers in paddy soils than AOB in our study. Our results were in accordance with previous studies, which showed that AOA were the dominant group among ammonia-oxidizing prokaryotes in paddy soil [47, 48]. Furthermore, some researchers have previously indicated that AOA are the main drivers of ammonia oxidation in soil by measuring transcriptional activity of AOA *amoA* genes or based on analysis with stable isotope probe [49, 50]. Correlation analysis illustrated that soil total C, total N, C/N ratio, pH, and ammonium content were correlated with abundance of AOA rather than AOB. This suggests that AOA were more sensitive to soil characteristics than AOB, which is consistent with the findings of previous studies [51, 52]. Our study indicated that environmental factors might be important in affecting the relative abundances of AOA and AOB.

Denitrification is of major importance to the N cycle and consists of four reaction steps in which nitrate is reduced into dinitrogen gas. The reduction of nitrite to nitric oxide is catalyzed by two structurally different nitrite reductases (Nir): one contains copper (Cu-Nir) encoded by the *nirK* gene while the other contains heme c and heme d1 (cd1-Nir) encoded by the *nirS* gene [53]. In this study, denitrification genes (*nirS* and *nirK*) decreased significantly with soil depth at all three sites. Both genes accounted for the largest proportion of measured N-cycling genes, which is in accordance with previous findings that their proportions in bacterial community ranged from 0.5 to 5% [54, 55]. In our study, the abundance of *nirS* was greater than that of *nirK* in 0–20 cm surface paddy soil, but in subsoil *nirK* abundance was higher than *nirS*, which suggests that *nirK*-harboring denitrifiers thrived in the deeper soil layers rather than *nirS*-harboring denitrifiers. Correlation analysis illustrated that total C, total N, soil water content, C/N ratio, pH, DOC concentration, and sand and clay content were correlated with *nirK* or *nirS* abundances among soil properties, and soil pH and sand content had the strongest correlations with *nirK* gene abundance (*P* < 0.01). Previous studies have shown that *nirS* denitrifiers are located mostly in the rhizosphere, whereas *nirK* denitrifiers are more abundant in the bulk soil [56, 57]. This suggests that soil nutrient resource availability is an important factor influencing the denitrifier community. Clark et al. (2012) found that soil properties (soil pH, clay, and C and N contents) had a major influence on denitrifier communities [58]. In addition, Bárta et al. (2010) found *nirK* gene abundance was higher in more alkaline soils and *nirS* abundance was higher in more acidic soils [59]. Dini-Andreote et al. (2016) indicated that *nirK* gene abundance was positively and significantly correlated with soil pH and sand content (p = 0.70 and 0.69, respectively; *P* < 0.01) [60]. In this study, resource availability, pH and soil physical properties showed significant changes along the soil depth gradient or among the three regions, which might represent an important effect on the distribution of *nirS* and *nirK*-containing denitrifiers.

### Linking the environmental factors and N-cycling abundance

In our study, not only soil properties, but also climate parameters were significantly correlated with N-cycling gene abundances. Previous studies have indicated that soil physicochemical changes are important factors influencing the distribution of N-cycling functional groups including pH [61, 62], dissolved oxygen levels [59, 63], nutrient resource [60, 61], salinity [64], C/N ratio [44, 61], and soil texture [58, 65]. However, there has been little research focusing on the distribution of N-cycling microbes at a large scale. Hu et al. (2015) found that the biogeographic distributions of both AOA and AOB were significantly regulated by spatial factors (latitude), climatic factors (mean annual temperature and precipitation), and geochemical factors (soil pH, total N, sulfate, clay%, and C/N ratio) [66]. Bru et al. (2011) reported that climate explained around 10–13% of the variability in the relative abundances of *nirS* and *nirK* denitrifiers within the total bacterial community when the distribution of N-cycling functional microbial communities was investigated over 107 sites in Burgundy, a 31,500 km^2^ region of France [55]. In this study, we found that climate parameters and elevation were significantly correlated with AOA, *nirK*, and *nirS* gene abundances at the large scale, which, together with soil properties, affected the changes of N-cycling gene abundances.

In this study, we used q-PCR method to characterize the changes of microbial populations and N-cycling gene abundances in the paddy soil profile. However, this method only detected the abundances of specific genes that we have designed to target. In the future, more novel metagenomics and metatranscriptomics approaches will enable us to connect specific bacterial and fungal populations to specific nitrogen cycling processes.

## Conclusions

In this study, we examined changes of microbial populations (16S rRNA and 18S rRNA) and five key N-cycling gene abundances along three soil profiles in paddy fields. Our results showed that the abundances of microbial and N-cycling functional genes (except AOA) generally decreased significantly along the soil depth gradient; however, the AOA were enriched in the deeper soil layers (20–40 cm). The abundance of AOA genes was higher than those of AOB throughout the paddy soil profile, and the abundances of *nirS* and *nirK* genes were dominant in topsoil and deeper soil, respectively. Soil properties and climate parameters had significant relationships with N-cycling gene abundances, which should be explored further to assess their roles in N cycling for applications in sustainable agriculture and environmental management.
